# Pre-Implantation Mouse Embryos Cultured In Vitro under Different Oxygen Concentrations Show Altered Ultrastructures

**DOI:** 10.3390/ijerph17103384

**Published:** 2020-05-13

**Authors:** Manuel Belli, Paolo Rinaudo, Maria Grazia Palmerini, Elena Ruggeri, Sevastiani Antonouli, Stefania Annarita Nottola, Guido Macchiarelli

**Affiliations:** 1 Department of Life, Health and Environmental Sciences, University of L’Aquila, 67100 L’Aquila, Italy; manuel.belli@univaq.it (M.B.); mariagrazia.palmerini@univaq.it (M.G.P.); sevastiani.antonouli@graduate.univaq.it (S.A.); guido.macchiarelli@univaq.it (G.M.); 2 Center for Reproductive Sciences, Department of Obgyn, University of California San Francisco, San Francisco, CA 94143, USA; elenaruggeri85@gmail.com; 3 Department of Anatomy, Histology, Forensic Medicine and Orthopaedics, La Sapienza University of Rome, 00161 Rome, Italy; stefania.nottola@uniroma1.it

**Keywords:** IVF, oxygen concentration, embryo, in vitro culture, TEM

## Abstract

Assisted Reproductive Technologies routinely utilize different culture media and oxygen (O_2_) concentrations to culture human embryos. Overall, embryos cultured under physiological O_2_ tension (5%) have improved development compared to embryos cultured under atmospheric O_2_ conditions (20%). The mechanisms responsible for this remain unclear. This study aimed to evaluate the effect of physiologic (5%) or atmospheric O_2_ (20%) tension on the microscopic ultrastructure of pre-implantation mouse embryos using Transmission Electron Microscopy (TEM). Embryos flushed out of the uterus after natural mating were used as the control. For use as the control, 2-cells, 4-cells, morulae, and blastocysts were flushed out of the uterus after natural fertilization. In vitro fertilization (IVF) was performed using potassium simplex optimized medium (KSOM) under different O_2_ tensions (5% and 20%) until the blastocyst stage. After collection, embryos were subjected to the standard preparative for light microscopy (LM) and TEM. We found that culture in vitro under 5% and 20% O_2_ results in an increase of vacuolated shaped mitochondria, cytoplasmic vacuolization and presence of multi-vesicular bodies at every embryonic stage. In addition, blastocysts generated by IVF under 5% and 20% O_2_ showed a lower content of heterochromatin, an interruption of the trophectodermal and inner cell mass cell membranes, an increased density of residual bodies, and high levels of glycogen granules in the cytoplasm. In conclusion, this study suggests that in vitro culture, particularly under atmospheric O_2_ tension, causes stage-specific changes in preimplantation embryo ultrastructure. In addition, atmospheric (20%) O_2_ is associated with increased alterations in embryonic ultrastructure; these changes may explain the reduced embryonic development of embryos cultured with 20% O_2_.

## 1. Introduction

Preimplantation embryos show an incredible ability to adapt to different culture conditions and result in viable offspring. However, it is becoming apparent that preimplantation embryos may respond differently to different culture conditions. For example, mouse embryos cultured in different media may result in offspring with altered intrauterine growth [1] or abnormal adult phenotypes [2,3].

In addition, the effect of different oxygen concentration (O_2_) is known to profoundly affect the development of preimplantation embryos. Overall, in vitro fertilization and culture has been performed in either 5% O_2_ (physiologic) or 20% O_2_ (atmospheric) [4,5]. There is an oxygen gradient from the fallopian tubes to the uterus, with most mammals having a lower O_2_ concentration in the uterus [6]. The O_2_ concentration in the oviducts of mammals has been found to range from 5% to 8.7%, while in the uterus ranges from 1.5% to 8% [6,7]. In mice, the O_2_ tension in the oviduct ranges from 2.7% to 8%, and 1.5% to 2% in the uterus [8,9]. Recently, the use of atmospheric oxygen tension to culture human embryos has decreased, although a significant percentage of clinics still use 20% O_2_ [4]. Many studies in several species have demonstrated that pre-implantation embryo development is improved by culturing embryos under a lower concentration of O_2_ [10,11,12], most likely because the physiological concentration of O_2_ in vitro corresponds to the O_2_ tension existent in the female reproductive tract [13,14]. Mammalian embryos cultured under a lower O_2_ tension (5%) showed higher cleavage, implantation, pregnancy and live birth rates [15,16]. However, how different oxygen tensions affect the morphology of pre-implantation mouse embryos cultured in vitro is not completely understood. Recently, we found that pre-implantation embryos cultured under 20% O_2_ showed a decreased number of normal-appearing mitochondria, an increase in vacuoles and an increase in mitochondria containing vacuoles, and were therefore considered abnormal compared to in vivo embryos. Vacuole formation is a sign of oocytes and pre-implantation embryo stress, frequently identified by electron microscopy (EM) [17]. In embryos, the presence of vacuoles is associated with decreased blastocyst formation [18]. In addition, the mitochondria of IVF-generated embryos had lower mitochondrial membrane potential and produced higher levels of reactive oxygen species (ROS) [5,19].

In this study, we used light (LM) and transmission electron microscopy (TEM) to describe the morphological changes occurring in pre-implantation mouse embryos (at the 2-cell, 4-cell, morula and blastocyst stage), following in vitro culture under physiologic (5%) and atmospheric (20%) concentrations of O_2,_ using in vivo embryos flushed out of the uterus as controls. Furthermore, we focused our analysis on the blastocyst stage, since embryo transfer at this stage is common, with increased occurrence of healthy singleton live birth [20,21]. These data will help us to understand how variations of O_2_ concentrations can affect, to what extent and at which stage, the ultrastructure of murine embryos cultured in vitro.

## 2. Materials and Methods

### 2.1. Chemicals

All materials were purchased from Sigma Chemical Co. (St. Louis, MO, USA) unless stated otherwise.

### 2.2. Animals

All experiments were approved by the Institutional Animal Care and Use Committee of the University of California, San Francisco (IACUC number AN153175-03F). All mice were kept in the University of California, San Francisco (UCSF) animal facility, with regulated temperature conditions of 21 to 23°C, a 12L:12D cycle, and ad libitum access to water and food. On average, to generate mice at each developmental stage, 5 females were mated with males (FB group) or eggs from 5 females were used to perform IVF with sperm from one male.

### 2.3. Embryo Collection, In Vitro Fertilization and Embryo Culture

In vitro fertilization was performed as previously described. For each experiment, 5 Carworth Farms (CF1) female mice (6 weeks old) were super-ovulated by injecting 5 IU PMSG and, 42–46 h later, 5 IU hCG as described [22]. In brief, oocytes were collected from the ampullae 13 h after hCG injection. Then, the oocytes were incubated in EmbryoMax HTF (Millipore, MR-070-D) and sperm obtained from the capacitated cauda epididymis of male B6D2F1/J mice (8–9 weeks) for 4 h. The fertilized oocytes were washed and cultured in EmbryoMax KSOM + AA with D-Glucose (Millipore, MR-106-D) up to the blastocyst stage under mineral oil (Vitrolife, #10029) in 5% CO_2_ in humidified air with 20% oxygen (IVF 20% group), or 5% CO_2_ and 5% oxygen at 37 °C (IVF 5% group). Control embryos (C) at different phases of development were flushed out of the uterus. Embryos in 2-cells, 4-cells, morula and blastocyst (n = 5/stage) stages were analyzed in triplicate.

For each experiment, 5 CF1 female mice (8–9 weeks) were super-ovulated by injecting 5 IU PMSG and, 42–46 h later, 5 IU hCG. The CF1 females were then mated to B6D2F1/J males. The next morning, after the vaginal plug was identified (the presence of the plug was considered at day 0.5 of pregnancy -E0.5), the uteri were removed and placed in a petri dish, and each horn was flushed using a 1ml insulin syringe loaded with warm culture medium. The resulting flushed embryos were collected. The collections of various developmental stages from the IVF and control groups were based on the morphology of the embryos, and were about as follows: 2-cell, C-24–28 h, IVF 38 h; 4-cell, C-30–40 h, IVF 48-50 h; morula, C-60–70 h, IVF 75–85 h; blastocyst, C-82–84h, IVF 108–110 h. Table 1 shows a schematic representation of the experimental design. Five embryos of each developmental stage (2-cells, 4-cells, morula and blastocysts) in 3 biological replicates were collected.

### 2.4. Light Microscopy (LM) and Transmission Electron Microscopy (TEM)

Five embryos for each developmental stage were collected in triplicate, washed in PBS and immediately fixed in 2.5% glutaraldehyde (Agar Scientific, Cambridge Road Stansted Stansted Essex, United Kingdom) in phosphate-buffered saline (PBS). Fixed specimens were stored at 4 °C for 2–5 days and then processed as previously described [23,24,25,26]. Samples were washed in PBS and postfixed with 1% osmium tetroxide (Agar Scientific, Stansted, UK) in PBS, and rinsed again in PBS. Each embryo was then embedded in small blocks of 1% agar of about 5 × 5 × 1 mm in size, dehydrated in an ascending series of ethanol, treated with propylene oxide for solvent substitution, embedded in epoxy resin EMbed-812 (Electron Microscopy Sciences, 1560 Industry Road, Hatfield, PA, USA) and sectioned using a Reichert–Jung Ultracut E ultramicrotome. Semithin sections (1 mm thick) were stained with Toluidine Blue, examined using LM (Zeiss Axioskop, Zeiss, Oberkochen, Germany) and photographed using a digital camera (Leica DFC230, Wetzlar, Germany). Ultrathin sections (60–80 nm) were cut with a diamond knife, mounted on copper grids, and contrasted with saturated uranyl acetate and lead citrate (SIC, Rome, Italy). They were examined and photographed using Zeiss EM10 and Philips TEM CM100 Electron Microscopes operating at 80 kV. In brief, a grid was inserted into the TEM column, then a specific sample area was selected and micrographs (at low and high magnification) were acquired [27,28,29]. The following parameters were evaluated by LM and TEM and taken into consideration for qualitative assessment of the ultrastructural preservation of the embryos: general features (shape and dimensions); integrity of the cell membrane; type and quality of the organelles; characteristics of the nucleus; and presence and extent of cytoplasmic vacuolization [17]. Of note, we did not quantify the amount of chromatin (heterochromatin vs euchromatin) or lipid and glycogen content, but only performed a qualitative analysis of relative abundance using multiple embryo micrographs of equivalent magnification.

## 3. Results

### 3.1. 2-Cell Stage

**Figure 1:**
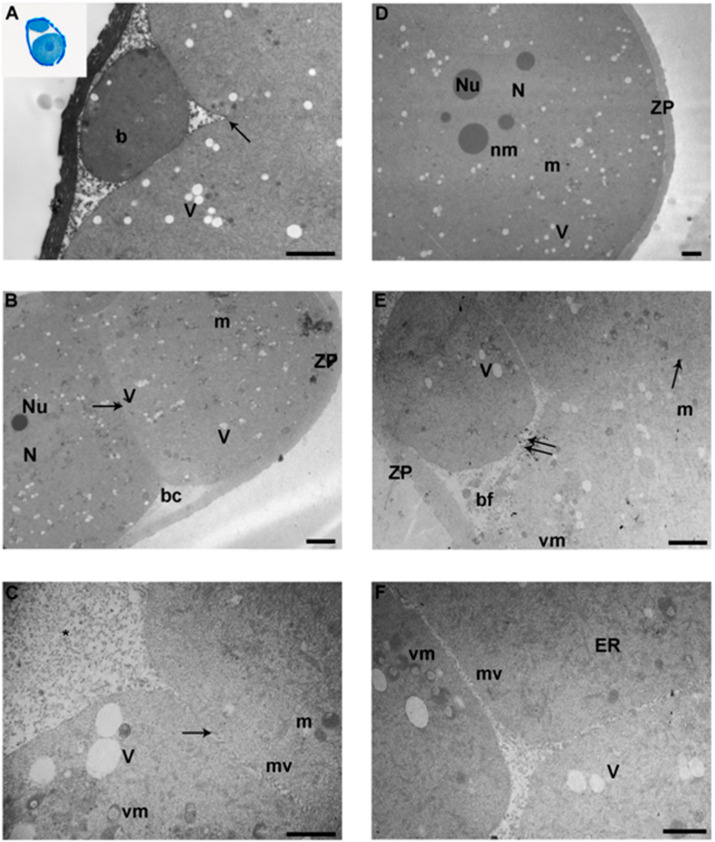
**Ultrastructural evaluation of 2-cell and 4-cell embryos. A–C. Representative micrographs of 2-cell embryo groups. A.** Transmission electron microscopy (TEM) micrograph of a 2-cell embryo of the IVF-5% group, showing a small blastomere forming (b). Arrows indicate the intercellular contacts (TEM. Bar: 2 μm). **Inset in A.** A representative image of a semithin section of a 2-cell embryo (LM. Mag: 40x). **B.** A representative picture of in vitro fertilized (IVF)-20% 2-cell embryo with evident nucleus (N) and nucleolus (Nu). Different blastomeres with cellular junctions (arrow) are visible (TEM. Bar: 4 μm). **C.** High magnification of IVF-20% 2-cell embryo adjacent blastomeres showing continuous intercellular contacts (arrow). Abundant cellular debris (*) is present in the inter-blastomeric space (TEM. Bar: 1 μm). **D–F**. Representative micrographs of 4-cell embryo groups. **D.** TEM micrograph of IVF-5% 4-cell embryo with evident nucleus (N) and nucleoli (Nu). Several vacuoles (V) and mitochondria (m) are evident. (TEM. Bar: 2 μm). **E.** Representative picture of IVF-20% 4-cell embryo showing numerous vacuoles (V), vacuolated mitochondria (vm) and blastomeric fragments (bf). Double arrows indicate the interruption of the intercellular contacts between blastomeres (TEM. Bar: 2 μm). **F.** TEM micrograph showing the inter-blastomeric cleft between 3 cells of IVF 20% (TEM. Bar: 1 μm). bc: blastocoel cavity; m: mitochondria; V: vacuoles; ZP: zona pellucida; vm: vacuolated mitochondria; mv: microvilli; arrow: intercellular contact; N: nucleus; nm: nuclear membrane; ER: endoplasmic reticulum.

By LM, 2-cell embryos cultured in vitro under 5% and 20% oxygen tension presented morphological features similar to the control 2-cell embryos. Blastomere were well-preserved, and the zona pellucida had a medium thickness of 2 µm. The cytoplasm showed several vacuoles in all groups (Figure 1A, inset).

By TEM, blastomeres of all the groups appeared well preserved. Blastomeres were spherical or ovoid and of equal size, with large, rounded nuclei (Figure 1B). The blastomeres were closely connected through immature cell contacts situated along their radial walls (Figure 1A–C). Inter-blastomeric clefts and central cleavage cavities were already evident. Most embryos presented a few cytoplasmic fragments or cellular debris (Figure 1C). The organelles tended to concentrate in the proximity of the nuclear area. In the cortical area, organelles were rare (Figure 1A–C). The plasma membranes were folded in numerous thin microvilli (Figure 1C).

Differently from the in vivo control embryos, blastomeres of the 2-cell embryos cultured in vitro (both 5% and 20% O_2_) showed dilation of the SER and Golgi membranes, resulting in: 1) evident vacuolization of the cytoplasm, and 2) progressive vacuolization of the mitochondria, caused by the swelling and blebbing of their membranes (Figure 1A–C).

Mitochondria in all the groups appeared round or ovoid in shape. In this developmental phase, mitochondria seemed regularly distributed throughout the cytoplasm. Mitochondria showed an inert-looking aspect, with a dense matrix and a few arch-like cristae located peripherally or transversely (Figure 1B,C). Mitochondria were frequently found in the proximity of vacuoles in all the groups.

Vacuolated mitochondria, recognizable by membrane-bound fragments dispersed in the organelle surface, appeared to be more abundant in the IVF 20% group (Figure 1C). Rare mitochondria were duplicating or fusing (not shown).

### 3.2. 4-Cell Stage

At the 4-cellstage, embryos showed well-preserved blastomeres, as seen by LM analysis. The zona pellucida had a medium thickness of 1.5 µm, but several areas of thinning were visible (especially in the control and IVF-20% groups) (not shown).

TEM micrographs showed blastomeres of all the groups had a round-to-ovoid shape with large roundish nuclei, delimited by an intact electron-dense nuclear membrane. Chromatin was uniformly distributed. Nucleoli were frequently found in all groups (Figure 1D). Microvilli extending from the plasma membrane into the peri-vitelline space were found in all groups. Most embryos presented a few cytoplasmic fragments or cellular debris (Figure 1E). Organelles appeared less numerous than in the 2-cell stage, and were irregularly distributed in the cytoplasm. Some blastomeres, in all groups, showed a preferential localization of organelles in the perinuclear zone.

SER was tubular in appearance in all groups. Mitochondria were spherical or oval, showed dense granular matrices and presented a few arch-like cristae. Compared to the 2-cell embryos, mitochondrial matrices were less dense, and had increased cristae. Several mitochondria were closely associated with SER elements. Mitochondria often showed evident vacuoles or dense bodies in their matrices (Figure 1E,F), particularly in the IVF 20 % group.

### 3.3. Morula

**Figure 2:**
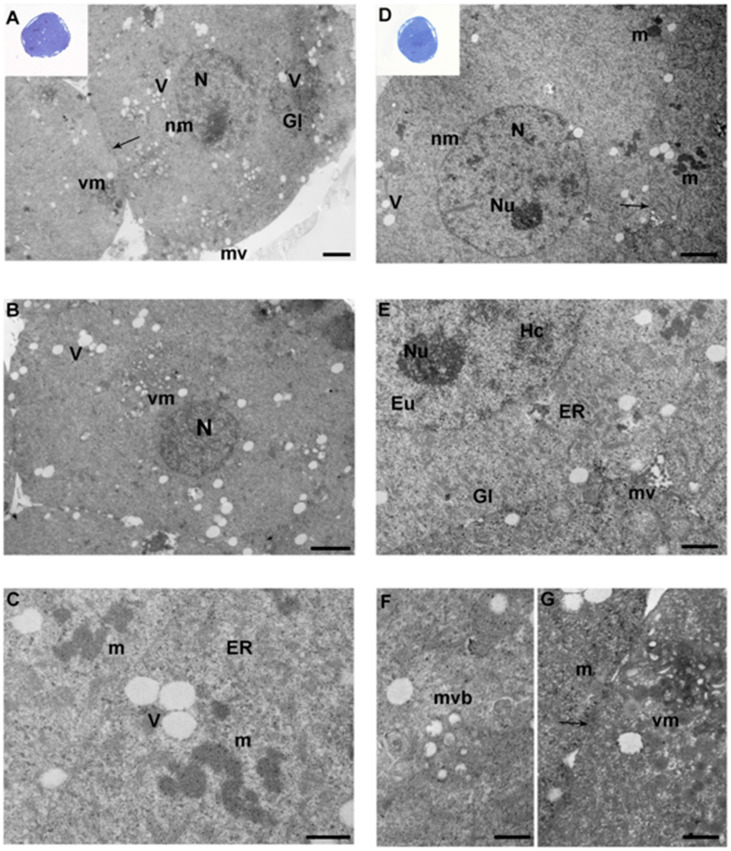
**Ultrastructural analysis of morulae. A–C. Representative micrographs of morulae from the control and IVF 5% groups, showing similar morphological features. A.** Ultrastructure of the control group morula with large round nucleus (N). Arrow indicates the intercellular contacts. (TEM. Bar: 1 μm). **Inset in A:** a representative image of a semithin section of in vivo morula. Numerous blastomeres with well-stained nuclei are visible (LM. Mag: 40x). **B.** Morula showing numerous vacuoles (V) and vacuolated mitochondria (vm) (TEM. Bar: 2 μm). **C.** Representative picture of IVF-5% morula high electron-dense mitochondria (m) and vacuoles (V) (TEM. Bar: 0.6 μm). **D–G. IVF-20% morula ultrastructure. D.** Ultrastructure of IVF-20% morula with blastomeres in a compaction stage. Arrows indicate the intercellular contacts (TEM. Bar: 2 μm). **Inset in D.** A representative image of a semithin section of IVF-20% morulae (LM. Mag: 40x). **E.** High magnification of an IVF-20% morula nucleus (N) with evident nucleolus (Nu) and patches of hetero- (Hc) and euchromatin (Eu) (TEM. Bar: 1 μm). **F–G.** High magnification of an IVF-20% multivesicular body (mvb) (TEM. Bar: 0.6 μm) and vacuolated mitochondria (vm) (TEM. Bars: 0.6 and 0.8 μm). N: nucleus; Nm: nuclear membrane; V: vacuoles; m: mitochondria; vm: vacuolated mitochondria; mv: microvilli; Gl: glycogen granules; ER: endoplasmic reticulum; nu: nucleolus; arrow: intercellular contact; Hc: heterochromatin; Eu: euchromatin; mvb: multivesicular body.

When analyzed by LM, blastomeres of the morulae of the control and IVF 5% groups appeared well stained and preserved, with wide inter-blastomeric spaces (Figure 2A, inset). Differently, morulae of the IVF-20% group showed the presence of compacted blastomeres, with a polyhedral shape (Figure 2D, inset). Zonae pellucida (medium thickness: 1–2 µm) were intact in all the groups. The nuclei were round or ovoid in shape, delimited by integral nuclear membranes, and with evident nucleoli (Figure 2A,D, insets).

By TEM, in the control and IVF-5% groups, rounded blastomeres contained round or slightly oval nuclei, delimited by intact nuclear membranes. Inside the nucleus, chromatin was dispersed with patches of heterochromatin and euchromatin, with clearly noticeable nucleoli (Figure 2A,B,E).

Differently to the other groups, the IVF-20% group showed compacted blastomeres of polyhedral and irregular shape, containing round/oval nuclei. Both external and internal cells showed a peculiar polygonal shape (Figure 2D,E).

In all groups, but especially in IVF embryos, several cells showed different grades of electron density. Intercellular contacts were more common compared to the previous developmental stages, with the appearance of specialized junction, especially at the apical limits of the outermost cells. Microvilli appeared larger and more abundant than in the previous stage, and projected into the perivitelline space and into the cleavage of the intercellular clefts (Figure 2A,E). Remnants of fibrillar lattice and glycogen granules were also seen (not shown). Numerous vacuoles and isolated glycogen granules were frequently found in the cytoplasm of all the groups (Figure 2A,E). Importantly, multi-vesicular bodies were found only in IVF 20% group (Figure 2F).

Irregularly shaped (tubular or round) mitochondria were generally distributed around the nuclei and, frequently, close to vacuoles. Mitochondrial electron-density was higher than in other embryo stages (Figure 2C). Numerous vacuolated mitochondria were present in all the experimental groups (Figure 2A,B,G).

### 3.4. Blastocyst

**Figure 3:**
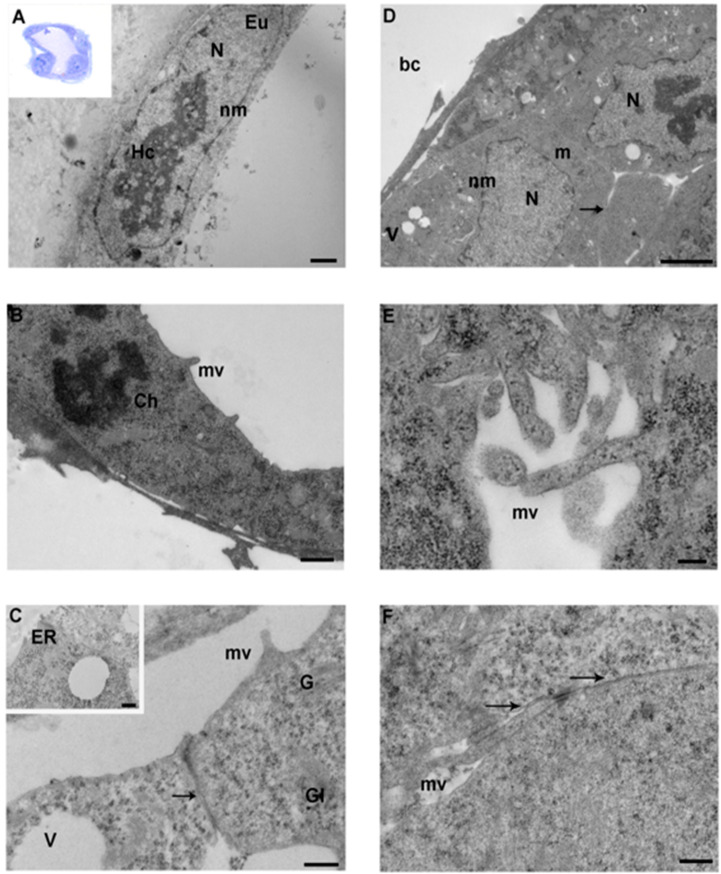
**Ultrastructure of a control group blastocyst. A.** TEM micrograph of TE cell with a large nucleus (N). The cell showed a clear nuclear content of heterochromatin (Hc) and euchromatin (Eu) (TEM. Bar: 1 μm). **Inset in A**. A representative image of a semithin section of a whole blastocyst (LM. Mag: 40x). **B.** TE cell with evident chromosomes (Ch) (TEM. Bar: 1 μm). **C.** High magnification of intercellular junction (arrow) (TEM. Bar: 0.4 μm). **Inset in C.** Details of endoplasmic reticulum vesicle (ER) and vacuole (V). (TEM. Bar: 0.4 μm). **D.** Ultrastructure of ICM. Nuclei (N) show patches of heterochromatin (Hc) and euchromatin (Eu) (TEM. Bar: 2 μm). **E.** High magnification of microvilli (TEM. Bar: 0.2 μm). **F.** High magnification of intact intercellular junction (TEM. Bar: 0.4 μm). N: nucleus; Hc: heterochromatin; Eu: Euchromatin; nm: nuclear membrane; mv: microvilli; V: vacuoles; Gl: glycogen granules; arrow: intercellular contacts; bc: blastocoel cavity.

Blastocysts, as examined by LM, appeared roughly spherical, with well-preserved nuclei, and showed an amorphous zona pellucida (thickness 1–1.5 µM) (Figure 3A, inset).

**Figure 4:**
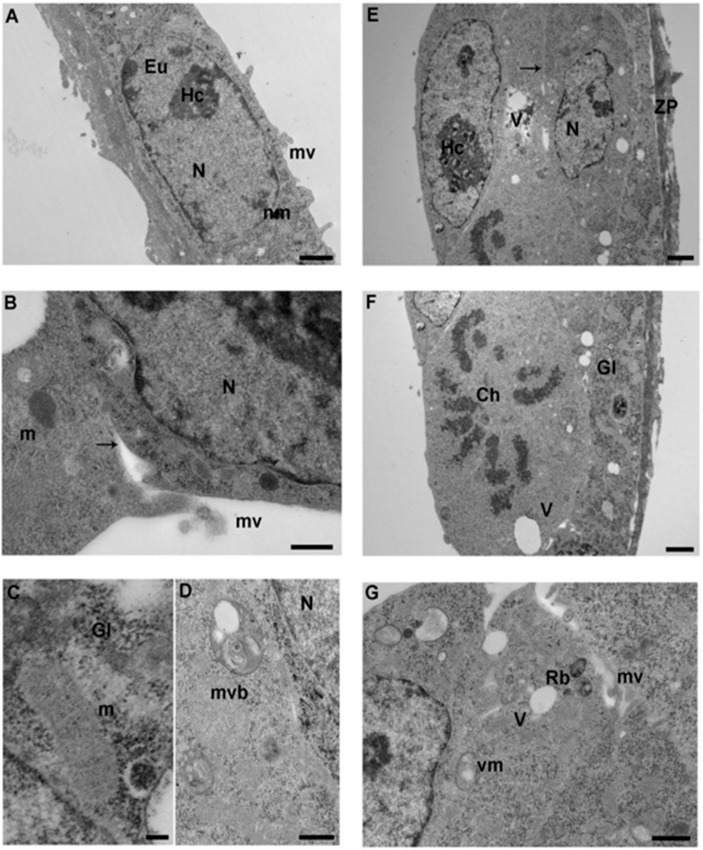
**Ultrastructure of IVF-5% blastocyst. A.** TEM micrograph of a TE cell with a large nucleus (N). (TEM. Bar: 1 μm). **B.** Intercellular junction (arrow) between TE cells (TEM. Bar: 0.6 μm). **C.** TEM micrograph showing mitochondria (m) with evident cristae (TEM. Bar: 0.1 μm). **D.** High magnification of a multi-vesicular body (mvb) (TEM. Bar: 0.6 μm). **E.** Ultrastructure of ICM cells showing ovoid nuclei (N) with patches of heterochromatin (Hc). The inner cell shows numerous vacuoles (V). The arrow indicates the intercellular contacts (TEM. Bar: 1 μm). **F.** ICM blastomere with evident chromosomes (Ch) (TEM. Bar: 1 μm). **G.** ICM cells with the presence of vacuolated mitochondria (vm) and residual bodies (Rb) (TEM. Bar: 0.8 μm). N: nucleus; nm: nuclear membrane; m: mitochondria; vm: vacuolated mitochondria; mv: microvilli; Hc: heterochromatin; Eu: euchromatin; Gl: glycogen granules; mvb: multivesicular body; ZP: zona pellucida; V: vacuoles; Rb: residual bodies.

**Figure 5:**
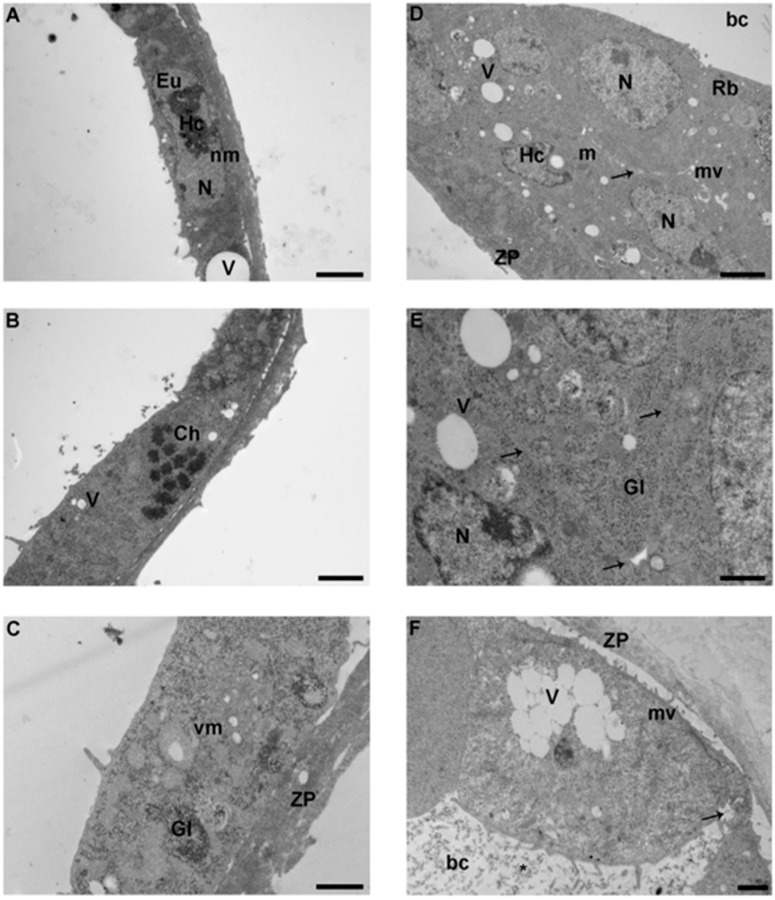
**Ultrastructure of IVF-20% blastocyst. A.** TE cells with large nuclei (N) and vacuoles (V). (TEM. Bar: 2 μm). **B.** TE blastomere in mitotic division with evident chromosome (Ch) (TEM. Bar: 2 μm). **C.** High magnification of TE blastomere showing vacuolated mitochondria and glycogen granules (TEM. Bar: 1 μm). **D.** Ultrastructure of ICM cells. Nuclei (N) showing a few patches of heterochromatin (Hc) (TEM. Bar: 2 μm). **E.** Intercellular contacts (arrow) between 3 ICM blastomeres (TEM. Bar: 1 μm). **F.** Extremely vacuolated blastomere exhibit interruption of the cellular membrane (arrow) and numerous cellular fragments (*) in the blastocoel cavity (bc) (TEM. Bar: 1 μm). N: nucleus; nm: nuclear membrane; Hc: heterochromatin; Eu: euchromatin; V: vacuoles; arrow: intercellular contacts; m: mitochondria; vm: vacuolated mitochondria; V: vacuoles; mv: microvilli; Gl: glycogen granules; Rb: residual bodies; ZP: zona pellucida; bc: blastocoel cavity.

By TEM, the cells of the trophectoderm (TE) formed a single continuous layer of flattened cuboidal polyhedral cells (Figure 3A, Figure 4A, Figure 5A). Cells of the inner cell mass (ICM) in proximity to the blastocoel cavity were flattened, while the remaining ICM cells were arranged in 2–3 cell layers overlaid by the TE (Figure 3D, Figure 4E, Figure 5D).

Cell membranes were intact in all the groups, but occasional interruptions were seen, in particular in the IVF 20% group (Figure 5F). In all the groups, microvilli projecting toward the zona pellucida, and in the blastocyst cavity were irregularly distributed on the apical surface. Control group embryos showed more areas of dense microvilli compared to the other groups (Figure 3E) and both inner cell mass and TE cells showed the presence of extensive regions of less dense, granular cytoplasm. The dense regions of cytoplasm showed a plethora of organelles, typically in a juxtanuclear and peripheral position. Generally, nuclei presented an intact membrane and dispersed chromatin with patches of heterochromatin. Chromatin was scattered in the nucleoplasm and condensed at the nuclear membrane. The nuclei often appeared elongated, with some invaginations. Few ovoid nucleoli were present (Figure 4A, Figure 5D). Few cells were found during mitotic division with well-defined dense chromosomes with bipolar spindle connected with polar centrioles with pericentriolar provisions (Figure 3B, Figure 4F, Figure 5B). Remarkably, IVF 20% nuclei showed less abundant heterochromatin (Figure 5D) than in vivo and 5% O_2_ embryos (Figure 3A, Figure 4E). Differently from the two IVF groups, the SER of controls embryos contained multiple strands (Figure 3C, inset).

The TE and ICM cytoplasm of IVF groups, compared to the in vivo group, showed a high content of glycogen granules in mono-particulate (Figure 4C, Figure 5C) form, and an extensive cytoplasmic vacuolization, particularly evident in the blastomeres of TE cells (Figure 5D,F).

Further, IVF 20% blastocysts displayed more remarkable changes. Blebbing of the cytoplasmic membrane toward the blastocoel cavity was noted (Figure 5F). Glycogen granules delimited by a membrane appeared clumped in small areas throughout the cytoplasm; of note, they were more abundant in the IVF 20% group.

Occasional multi-vesicular bodies and lysosome-like bodies were scattered in the cytoplasm, more frequently in the IVF 5% and 20% groups (Figure 4D). However, both TE and ICM cells of the IVF groups showed the presence of lipid droplets and several residual bodies constituted by undigestible materials of high electron density.

Mitochondria were of elongated/tubular shape in both TE and ICM cells (Figure 4C). These mitochondria showed well defined transverse cristae, a clear sign of increased metabolic activity. Vacuolated mitochondria were more abundant in the IVF 20% group (Figure 5C). Mitochondria distribution did not change among the groups. Table 2 compares the ultrastructural markers of quality between control-blastocysts and IVFs-blastocysts.

## 4. Discussion

This paper offers a detailed analysis of the ultrastructure of mouse pre-implantation embryos generated in vivo or in vitro, and cultured under physiological and atmospheric O_2_ tension.

We decided to focus our analysis on embryos cultured with the same medium but different oxygen concentrations because reduced O_2_ tension has resulted in improved development to the blastocyst stage [14]. The use of a low O_2_ concentration is beneficial for embryo development in vitro to the blastocyst stage in mice [12,19,30,31], hamsters [32], rabbits [33], pigs [34], sheep [35] and humans [16,36].

While transcriptomic analysis has revealed profound changes in gene expression in embryos cultured under different oxygen concentrations [37], the morphologic effect of oxygen on embryos is not well known, and was first described by our group [5]. To this end, the current detailed ultrastructural analysis provides a deeper understanding of events occurring during development [28,38,39,40,41], and allows us to establish a partial correlation between the observed phenotype and the transcriptomic assay, with clinical implications that are valuable in several fields [42,43,44,45].

Although the ultrastructural characteristics of mouse embryos have already been reported [46,47], this is the second time that pre-implantation mouse embryos cultured under different O_2_ were evaluated at the ultrastructural level by TEM [5].

We focused our morphological evaluation on blastocysts because, during IVF-ET, embryos are often transferred at the blastocyst stage. Recent studies demonstrated that, in fresh human IVF, the transfer of embryos at the blastocyst stage improved the ongoing clinical pregnancy rates, with optimal rates of clinical pregnancy, ongoing pregnancy and cumulative ongoing pregnancy [48,49].

Overall, in agreement with others [46,50,51], we found that both control and IVF groups’ embryos did not show major alterations in shape, size and general organization of the cytoplasm.

At the same time, we found that embryos generated and cultured in vitro resulted in several changes, observable at all developmental stages: (1) increase of vacuolated shaped mitochondria, (2) increase in vacuoles in the cytoplasm, (3) presence of multi-vesicular bodies, and (4) presence of advanced compaction in morulae in the IVF 20%.

These morphological data are in agreement with our previous results, thus confirming that embryo culture, in particular at 20% O_2_, may be responsible for altering mitochondrial morphology (i.e., the presence of vacuolated mitochondria) [5]. Table 3 summarizes the main results of our previous works. Vacuolated mitochondria are associated with fusion and fission processes, and unusual metabolic activity of the preimplantation embryo [52,53]. Importantly, the findings of lower numbers (i.e., density/area) of normal-appearing mitochondria and increased numbers of abnormal-appearing mitochondria were associated with alterations of mitochondrial function; in particular, reduced mitochondrial membrane potential and altered ATP production in IVF embryos [5]. Furthermore, the increase of vacuolization in IVF embryos and the presence of multi-vesicular bodies, particularly evident in the IVF 20% group, could indicate an increase in mitochondrial degeneration [5,54]. The presence of multi-vesicular bodies, associated with abundance of vacuoles in the cytoplasm of oocyte and pre-implantation embryos, is an unusual feature, and is considered a degenerative process determined by the swelling and combination of isolated SER vesicles [55] probably related to cytoskeletal alterations [56]. It is well known that mitochondria may be sensitive to high O_2_ concentration, and mitochondrial dysfunctions could represent the crucial factor predisposing to embryo developmental failure [57,58].

In addition, IVF blastocysts showed some other minor findings; specifically, deep invaginations in the nuclear surface and lower content of heterochromatin, and interruption to the TE or ICM cell membrane.

More importantly, heterochromatin was frequently observed in both ICM and TE nuclei of control and IVF 5% blastocysts, whereas the euchromatin was prominent in the IVF 20% blastocysts. Heterochromatin contains silent genes [59], while euchromatin is usually under active transcription [60]. These findings would indicate that IVF 20% embryos have increased transcriptional activity. This was shown in our prior publication, where the gene expression of embryos cultured under 20% oxygen was ten times more altered than in embryos cultured under 5% O_2_ [31,37]. For example, blastocysts cultured in KAA and 5% O_2_ showed 264 mis-expressed genes, while blastocysts cultured in KAA and 20% O_2_ showed a stunning 2133 altered genes. Pathways more commonly altered in the atmospheric oxygen conditions included RNA post translational modification, energy production, molecular transport, cell growth and organismal development [37]. Given these findings, future work should establish the epigenetic signature (DNA methylation and chromatin accessibility) of embryos cultured in vitro or in vivo. This is particularly important given that embryos undergo an important decline in DNA methylation during the preimplantation period, followed by re-methylation soon after the blastocyst stage [61].

The presence of more or less abundant nuclear invaginations is associated with nuclear size reduction. They reflect a mechanism for the adaptation of the nuclear envelope and its lamina to a shrinking nuclear size during preimplantation development [62].

Another important finding is that IVF embryos showed higher levels of glycogen than the control. Glycogen is physiologically present in the cytoplasm of the pre-implantation embryo. Biochemical and histochemical studies indicate that glycogen synthesis occurs mainly during the cleavage stage [63]. Cytoplasmic granules of glycogen represent an essential energetic store for the cleaving embryo. Therefore, the excessive accumulation of glycogen granules showed in IVF 20% blastocysts could be associated with a ‘block’ of glycogenolysis secondary to the mitochondrial alterations previously described. Since glycogen is an important energetic substrate used by mitochondria, these morphological findings suggest that the IVF procedure, and mainly high O_2_ concentrations, alter the normal metabolism of the developing embryos [12,64].

Among the limitations of this paper, several findings (relative amount of euchromatin vs heterochromatin, lipid content, glycogen content) were qualitative and not quantitative in nature. Future work should exactly quantify the relative abundance of each component.

Finally, IVF groups contained numerous residual bodies. The presence of these structures indicates increased cytoplasmic degradation and increased autophagy: in many cell types, autophagic vacuoles and related structures are the structures used for digestion of damaged organelles and mitochondria by the cell [65,66].

## 5. Conclusions

In conclusion, our results indicate that the stress of culture, especially in the presence of atmospheric O_2_, results in important morphological alterations. The morphological alterations found in IVF embryos reflect the reduced development of preimplantation mouse embryos during in vitro culture. We can further speculate that these morphological changes might be associated with significant changes in pre-implantation embryo metabolism.

Overall, our data support the interpretation that preimplantation embryos are vulnerable to the environmental disturbances induced by high O_2_ concentrations, and therefore optimal O_2_ exposure or the addition of an antioxidant in extended culture may be a key factor for improved embryo development.

## Figures and Tables

**Table 1:** Schematic representation of the experimental design.

Control: In Vivo Fertilized Mouse Embryos Flushed from the Uterus after Natural Fertilization	In Vitro Fertilization (IVF) 5%: IVF Performed in Optimal Conditions (Potassium Simplex Optimized Medium-KSOM with Amino Acids and 5% Oxygen)	IVF 20%: IVF Performed in High Oxygen (KSOM with Amino Acids and 20% Oxygen)
2-cell: 24–28 h	2-cell: 38 h	2-cell: 38-h
4-cell: 36–40 h	4-cell: 48–50 h	4-cell: 48–50 h
Morula: 60–70 h	Morula: 75–85 h	Morula: 75–85 h
Blastocyst: 82–84 h	Blastocyst: 108–110 h	Blastocyst: 108–110 h

**Table 2:** Comparison of the main ultrastructural marker of quality among blastocyst groups (control vs IVF 5% and 20%). Legends: N.D.: no differences.

	Blastocyst Control	IVF 5% Blastocyst	IVF 20% Blastocyst
Nuclear Shape	Oval or irregular shape. The surface showed minor irregularities.	Oval or irregular shape. The surface showed minor irregularities.	Oval, round or irregular shape. The surface presented minor irregularities and rare deep invaginations.
Nuclear envelope	Common structure formed by two intact membrane. Perinuclear space is often interrupted by nuclear pores.	N.D. (no differences)	N.D.
Hetero Chromatin	Regularly distributed in the nucleus with minor condensation close the nuclear envelope.	N.D.	Lower content of heterochromatin.
Nucleoli	One or two per nucleoli with irregular shape and reticular aspect.	N.D.	N.D.
Cell membrane	TE cell membrane appeared smooth with few and short microvilli. ICM cell surface presented numerous and longer microvilli.	N.D.	Interruption of the TE or ICM cell membrane were occasionally seen.
Mitochondria	Elongated and tubular in shape with long cristae and a medium dense matrix. Vacuolated mitochondria were present.	Higher density of vacuolated mitochondria.	Higher density of vacuolated mitochondria.
Vacuoles	Vacuoles were present but with smaller dimension if compared with those of the previous developmental stages.	Higher density of vacuoles.	Higher density of vacuoles.
Residual bodies	Occasionally seen in the cytoplasm.	Frequently seen in the cytoplasm.	Frequently seen in the cytoplasm. Smaller than the ones of the previous group.
Multi vesicular bodies	No presence of MVB in this group.	Occasionally found in the cytoplasm.	Occasionally found in the cytoplasm.
RER	Usually found in form of isolated cisternae.	N.D.	N.D.
SER	Present in the cytoplasm in form of small and smooth vesicles. Vesicles organized in cisternae are occasionally seen.	Cytoplasm showed lower density of SER vesicle.	Cytoplasm showed lower density of SER vesicle.
Golgi	Occasionally seen in the perinuclear region.	N.D.	N.D.
Glycogen	Present in the cytoplasm in mono-particulate form.	Higher density of glycogen granules in the cytoplasm.	Higher density of glycogen granules in the cytoplasm.
Lipid droplets	Lipid droplets were present quite regularly in the cytoplasm.	N.D.	N.D.

**Table 3:** Main alterations detected in prior studies of blastocysts cultured under physiologic (5%) and atmospheric (20%) O_2_ concentration, compared to in vivo blastocysts. The relative increase is based assuming 100% in vivo blastocysts.

	5% O_2_ Concentration	20% O_2_ Concentration	References
**Mitochondrial numerical density**	~100–200 less mitochondria/area detected.	~300 less mitochondria/area detected.	Belli et al., 2019 [5]
**Vacuolization**	No differences.	Nearly 200 more vacuoles/area detected.	Belli et al., 2019 [5]
**mtDNA copy number**	~10^7^ less copied detected.	~1.5 (10^7^) less copies detected.	Belli et al., 2019 [5]
**Alteration of the global gene expression**	The expression of 264 genes is altered compared to in vivo control.	The expression of 2133 genes is altered compared to in vivo control	Feuer et al., 2017 [37]
**Cell number**	25 more blastomeres than in vivo control.	10 less blastomeres than in vivo control.	Rinaudo et al., 2006 [31]
